# Supplementary figures and images for: Barriers to Medication Review Process Implementation—Cross-Sectional Study among Community Pharmacists in Jordan
Source: Healthcare (Basel). 2022 Mar 31;10(4):651. doi: 10.3390/healthcare10040651 (PMC9025090; doi:10.3390/healthcare10040651)

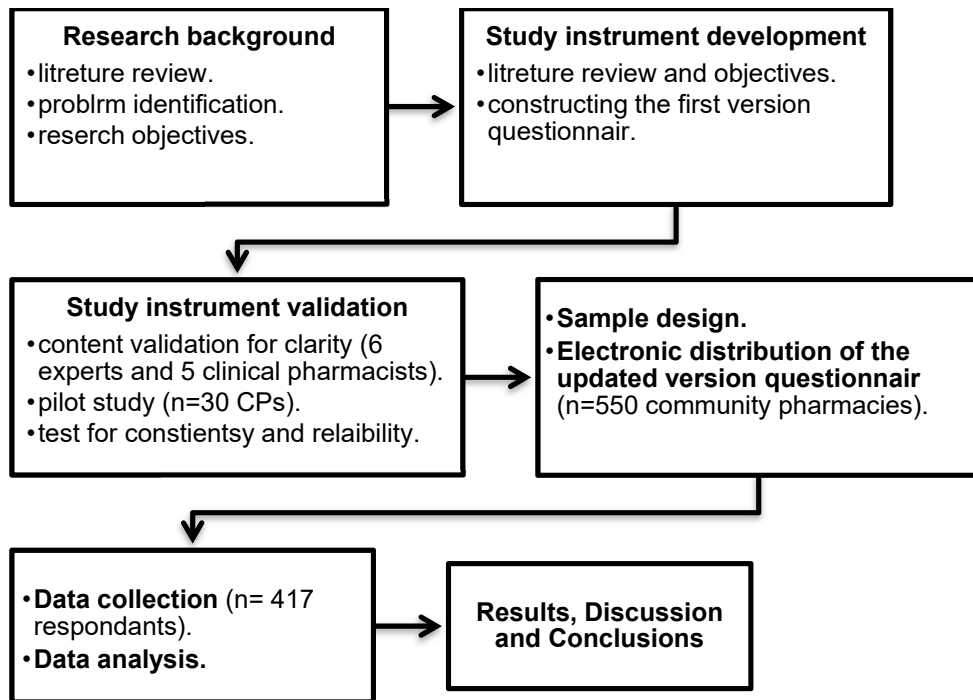

**Figure S1.** Flow chart of the whole study process. Abbreviations: CPs, Community Pharmacists

Supplement: Supplementary file 1 [file healthcare-10-00651-s001.zip › healthcare-1636093-supplementary.pdf]
